# Cataract management in children: a review of the literature and current practice across five large UK centres

**DOI:** 10.1038/s41433-020-1115-6

**Published:** 2020-08-10

**Authors:** J. E. Self, R. Taylor, A. L. Solebo, S. Biswas, M. Parulekar, A. Dev Borman, J. Ashworth, R. McClenaghan, J. Abbott, E. O’Flynn, D. Hildebrand, I. C. Lloyd

**Affiliations:** 1 Department of Ophthalmology, University Hospital Southampton, Southampton, UK; 2 Clinical and Experimental Sciences, School of Medicine, University of Southampton, Southampton, UK; 3 Department of Medical Genetics, St Mary’s Hospital, Manchester, UK; 4 Great Ormond Street Hospital, London, UK; 5 Manchester Royal Eye Hospital, Manchester, UK; 6 Birmingham Children’s Hospital, Birmingham, UK; 7 Oxford Eye Hospital, Oxford, UK

**Keywords:** Hereditary eye disease, Paediatrics

## Abstract

Congenital and childhood cataracts are uncommon but regularly seen in the clinics of most paediatric ophthalmology teams in the UK. They are often associated with profound visual loss and a large proportion have a genetic aetiology, some with significant extra-ocular comorbidities. Optimal diagnosis and treatment typically require close collaboration within multidisciplinary teams. Surgery remains the mainstay of treatment. A variety of surgical techniques, timings of intervention and options for optical correction have been advocated making management seem complex for those seeing affected children infrequently. This paper summarises the proceedings of two recent RCOphth paediatric cataract study days, provides a literature review and describes the current UK ‘state of play’ in the management of paediatric cataracts.

## 儿童白内障的管理: 文献综述以及横跨英国五大临床中心当前的临床实践

先天性白内障和儿童白内障并不常见, 但在英国大多数儿科眼科团队的诊所里经常见到。它们通常与严重的视力丧失有关, 而且大部分与遗传因素有关, 有些还伴有明显的眼外合并症。最优化的诊断与治疗通常需要多学科团队的密切合作。

手术仍是当前治疗的主要手段。对于不常见到患病儿童的医生, 目前一直提倡的各种手术技术、干预时机及光学矫正的选择, 管理似乎有些复杂。

本文总结了英国皇家眼科医师协会年会最近的两个儿童白内障研究的进展, 完成了一篇文献综述, 并描述了目前英国在儿童白内障管理方面的“工作状态”。

## Introduction

The global prevalence of congenital cataract (CC) is estimated as between 2.2/10,000 and 13.6/10,000 [1]. Variation in prevalence between populations is likely due to better identification rates in countries with screening programmes (for both cataract and disorders linked to cataract), rubella immunisation rates and differing population genetics [1]. Similarly, the necessity for treatment varies, for example between dense cataracts present at birth, partial cataracts at birth or developmental cataracts which may progress during childhood. Early identification, diagnosis and appropriate clinical care are key to achieving optimal outcomes. Ideal management of children with cataract typically involves a team of healthcare professionals. Well-established clinical networks and referral pipelines are also key to optimum outcomes. From diagnosis to surgical techniques, management has changed significantly in recent years but there remains variation in practice in the UK. In this paper we review the literature and present consensus from five large specialist centres in the UK to help clinicians manage children with this rare but important condition.

## Before surgery

### When, and how urgently should a child with cataract be referred?

**Fig. 1:**
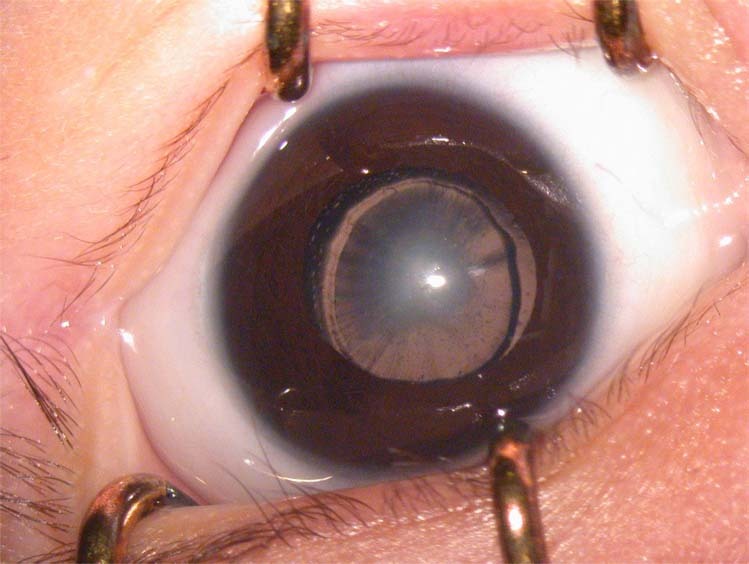
Dense congenital cataract. This image shows a dense nuclear cataract in a 6-week old infant.

Untreated dense CC (Fig. 1) leads to irreversible neurophysiological changes and sensory deprivation amblyopia. Associated adverse outcomes such as nystagmus and strabismus commonly co-exist [2]. Therefore, it is imperative that affected infants are referred promptly to centres able to manage them appropriately.

The UK National screening committee recommends that all eligible neonates are offered the NHS newborn and infant physical screening examination (NIPE). This should be carried out within 72 h of birth and again at 6–8 weeks of age [3]. The main purpose of the eye screening part of NIPE is to detect congenital ocular abnormalities of which CC is an important finding. The NIPE guidance currently states that infants screening positive at birth should be seen by an ophthalmologist by 2 weeks of age for confirmation of the finding and subsequent appropriate management. The 6–8 week examination takes place in a community setting and is usually carried out by a General Practitioner. NIPE guidance currently recommends that any associated risk factors are noted. These include family history of hereditary or CC, Trisomy 21, prematurity and maternal exposure during pregnancy to viruses such as rubella or CMV. Recommendations regarding the appearance of the eyes and visual behaviour is also provided. Lack of “red reflex” on photographs is flagged as a possible sign of lens opacity. The guidance recommends infants failing the 6–8 week examination should be examined by an ophthalmologist by 11 weeks of age [3]. However, optimal surgical results require very early referral and intervention. The best outcomes in dense unilateral CC follow surgery and optical correction before 6–8 weeks of age [4]. More recent studies of dense bilateral CC suggest that visual outcomes follow a linear model, correlating to length of visual deprivation, but that best results occur in those infants operated on by 8 weeks of (corrected) age [5–7]. This is at odds with the pragmatic NIPE recommendation of examination by an ophthalmologist by 11 weeks of age. Infants presenting this late tend to do less well [5].

A recent study from Cambridge found that fewer than 50% of the 33 children requiring cataract surgery under the age of 3 years of age were referred before 9 weeks of age and only 10 after an abnormal NIPE examination [8]. The authors found that the sensitivity of the NIPE examination, requiring the skilled use of a handheld direct ophthalmoscope, is relatively poor.

Infant eyes are very different to adult eyes. They are smaller and in those with cataract may be significantly microphthalmic. They have a hypoplastic and vascular iris, may exhibit significant microcoria, have an immature trabecular meshwork, a shallow anterior chamber (AC) and lack scleral rigidity. Infantile cataract surgery should thus ideally be performed by an experienced paediatric cataract surgeon within a team setting with concurrent expert paediatric anaesthesia, post-operative paediatric medical and nursing expertise and with availability of appropriate investigations and medical interventions [2].

Thorough pre-operative management is imperative. The majority of paediatric cataracts in the developed world have a genetic basis, with the largest group exhibiting autosomal dominant inheritance [9]. Thus ocular examination of parents and other family members can provide useful evidence of phenotypic variability. A small but significant proportion of infants and children presenting with bilateral cataract have an underlying systemic or metabolic disorder [9]. Thus, clinically informed biochemical investigations in combination with genetic testing are important. This concept is further discussed below.

In summary, centres providing paediatric cataract care should have an established expert team of experienced clinicians, optometrists and orthoptists to be able to provide tertiary level ophthalmological care. Children with cataract should be referred to such services promptly, within weeks or days, especially for infants with dense cataract in whom optimal surgery is within the first 2 months of life.

CLINICAL TIP: Surgery for infants with visually significant cataract is best performed in the first few weeks of life. Visual outcomes decline rapidly thereafter, thus infants identified with possible CC by non-specialist screening procedures should be referred urgently to specialists to confirm diagnosis. Subsequent referral to a specialist paediatric cataract service should be considered similarly urgent.

### Diagnostic workflow for children with CC

Whilst identification and timely surgical intervention in infants and children is crucial for preservation of sight, precise diagnosis is also important. CC is a highly heterogeneous disorder associated with a number of systemic diseases. Aetiologies may include trauma, maternal TORCHS (toxoplasmosis, rubella, cytomegalovirus, herpes simplex and syphilis) infection, intrauterine chemical or drug exposure, biochemical disturbance and genetic variation (chromosomal abnormalities or single gene mutation associated disorders). Pinpointing a diagnosis, even with the use of clinical algorithms, is complicated, [2, 10] and often protracted. Historically clinicians have pursued biochemical, genetic, clinical and imaging tests either simultaneously or consecutively and iteratively. This approach depends on accurate clinical phenotyping, involves many clinical professionals and numerous appointments, at significant cost to patients and healthcare services, yet yields a poor diagnostic rate [11]. Musleh et al. examined diagnostic rate in a ‘traditional’ investigative work-up of 27 consecutive bilateral CC referrals. An extremely low yield of 3.4% (*n* = 1) was found [12].

It has been estimated that at least 50% of CC cases are attributable to genetic abnormalities [13]. In the UK, this is likely an underestimate. All modes of inheritance have been demonstrated, though transmission as a dominant trait is most frequent [13]. Isolated CC may arise from mutations of lens specific genes such as those that encode crystallin, beaded filament or connexin proteins, leading to disruption of lens protein conformation, cellular organisation of the lenticular mass, or lens homoeostasis, respectively [14, 15]. CC may also occur as an early manifestation of multi-systemic conditions, [16, 17], with significant non-ophthalmic associations. More than 100 genes have now been associated with CC [18]. This extreme clinical and genetic heterogeneity represented a significant barrier to diagnosis until a decade ago when DNA sequencing methodologies evolved enabling the testing of multiple genes in a single genetic test [19, 20]. So called ‘Next Generation Sequencing’ (NGS) assays can be custom designed to accurately and rapidly screen a ‘panel’ of genes associated with a particular disease or phenotype [21], all known coding regions of the genome (exome) [22], or the entire genome, thus revolutionising genetic screening for heterogeneous diseases.

Recent studies have demonstrated the efficiency of gene panel NGS testing in CC diagnosis with impressive mutation pick-up rates, ranging from 70 to 85% for isolated cataract [23, 24], and 63% for syndromic CC [23, 25]. Moreover, these studies have highlighted the enormous clinical utility of ascertaining the precise cause of CC. Examples of this include where genetic diagnosis has [1]: altered the clinical hypothesis regarding predicted mode of inheritance, thereby re-defining recurrence risk and informing genetic counselling [2]; directed clinical management and patient care via pre-symptomatic diagnosis of significant multi-systemic disease [3]; diagnosed an unsuspected metabolic disease that is amenable to treatment where early treatment significantly reduces morbidity. Furthermore, a study of 50 patients by CC panel testing found that over 15% of cases were due to mutations in genes associated with inborn errors of metabolism [25]. Significantly, five of the six diagnosed conditions were amenable to treatment either by dietary management (e.g. stomatin-deficient cryohydrocytosis caused by mutation of *SLC2A1* treated by ketogenic diet) or therapeutic intervention (e.g. cerebrotendinous xanthomatosis due to mutation of *CYP27A1* treated with chenodeoxycholic acid and statins). This work recognises a poorly defined and likely under-diagnosed group of disorders for which congenital or childhood-onset cataract is an early clinical indicator highlighting a subgroup of patients that would benefit the most from prompt diagnosis by CC panel testing [25].

**Fig. 2:**
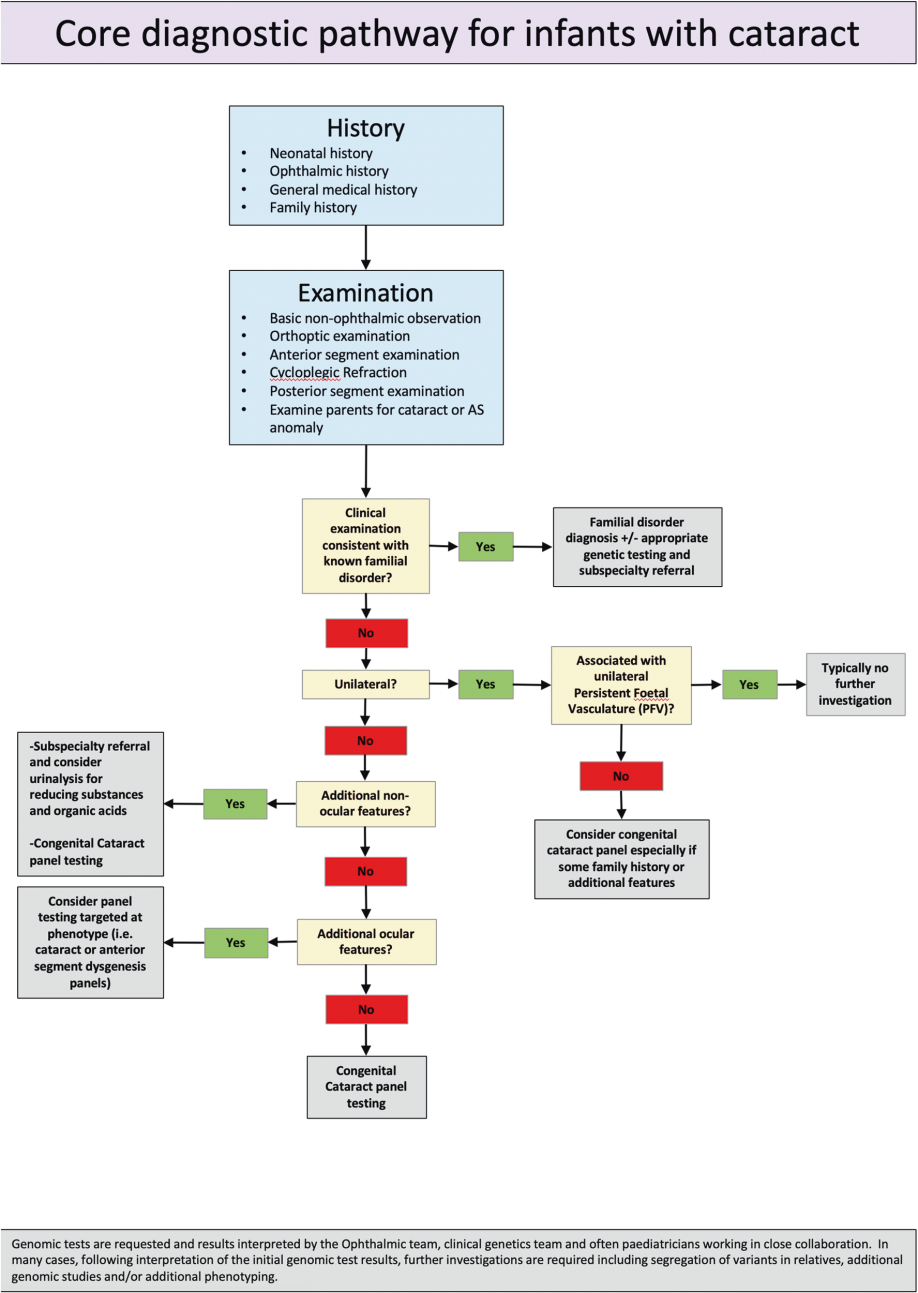
Diagnostic workflow for infants with congenital cataract. This figure depicts a typical diagnostic workflow for infants with congenital cataract.

Successful translation of CC gene panel testing into the clinical care pathway of CC patients has been shown to streamline management and reduce time to diagnosis from years to weeks [23, 25, 26], removing uncertainty around disease recognition and the need for multitudinous clinical tests of low diagnostic yield. Where genetic testing in readily available, it should be noted that this approach demands effective multidisciplinary team (MDT) working i.e. between geneticists, ophthalmologists, paediatric clinicians, clinical scientists, research experts and genetic counsellors as well as other clinical specialities where appropriate. Ordering a genetic test may become easier with centralisation of funding, but accurate interpretation of results and subsequent counselling of families relies on appropriate MDT infrastructure and experienced specialists. In Fig. 2 we provide a suggested workflow for CC incorporating NGS testing as the key diagnostic tool. However, it remains important to take a careful history, examine the child and family for ocular (and non-ocular) phenotypes and to collect evidence of relevant ante-, peri-, and post-natal problems. Ascertaining when cataracts (or any ophthalmic symptoms) were first noted is important both for management and provision of diagnostic clues (later-onset childhood cataracts may suggest metabolic disease). Identification of extra-ocular abnormalities should prompt sub-speciality referral for medical investigations in parallel to genetic testing and appropriate ophthalmic management. Furthermore, genomic tests are requested and results interpreted by the ophthalmic team, clinical genetics team and often paediatricians, working in close collaboration. In many cases, following interpretation of the initial genomic test results, further investigations are required including segregation of variants in relatives, additional genomic studies and/or additional phenotyping.

CLINICAL TIP: CC NGS panel testing offers a superior diagnostic yield than traditional diagnostic approaches [23] and is now used by all the authors and in most major centres in the UK.

### Planning for surgery: which operation and when?

#### Aphakia versus primary intraocular lens implantation

Cautionary tales of severe inflammation and poor visual outcome dissuaded paediatric ophthalmic surgeons from implanting intraocular lenses (IoLs) [27, 28], until surgical advances made it possible to retain a child’s capsule as support [29] enabling the use of a posterior chamber lens [30]. Implantation is now accepted practice for older children [31–33] but with children aged under 2 years, surgeons face the challenges of propensity to inflammation, immaturity and ocular size. Whilst the adult capsule measures 10.5 mm in diameter, the mean neonate capsule is considerably smaller at 7 mm, although it grows to 9 mm by 2 years of age and 10 mm by 5 years [29]. Primary IoL implantation in children under 2 years old does not appear to confer improved visual outcome over aphakic contact lenses (CLs). The Infant Aphakia Treatment Study (IATS) study group allocated 114 children aged less than 7 months old to either CL correction, or primary IoLs. Median vision at 5 years following surgery was the same in both groups (0.90 LogMAR) [34]. These findings were supported by the UK and Ireland ‘IoLunder2’ observational cohort study, which reported no visual benefit with implantation for children with bilateral or unilateral congenital or infantile cataract undergoing surgery in the first 2 years of life [5].

Despite initial speculation, IoLs have not been found to confer protection from the risk of secondary glaucoma. A recent meta-analysis which reported lower glaucoma risk in childhood pseudophakia was based on primary research limited by selection bias and failure to deal with confounding due to age at surgery [35]. Glaucoma prevalence at 5 years after surgery in the IATS was higher in the IoL group but this difference did not reach statistical significance [36]. IoLunder2 has also reported the absence of a protective effect with IoLs [5]. Wong et al. reported an increased risk of glaucoma following aborted IoL implantation necessitating explantation at primary surgery [37].

Visual axis re-opacification is much more common, and occurs earlier, following surgery with IoLs in children aged under 2 years versus aphakia [5, 38, 39]. This is due to the pro-inflammatory state of infancy and the scaffold effect of an intraocular foreign body [40]. Primary IoLs thus often commits the child, family and surgeon to secondary surgical procedure(s). Techniques such as ‘bag-in-lens’ implantation report much lower rates of re-operation [41], but have had limited uptake across the UK, USA and other settings [32, 33, 42, 43]. The consequence of further general anaesthetic for surgery to remove capsular opacity, with regards to potential negative impact on cognitive development, is unclear [44, 45]. For this reason, primary IoL implantation is currently not recommended as routine practise for children aged under 2 years. Clinicians who undertake implantation in infancy should counsel families on the risk of re-operation and balance this with the potential benefits of the avoidance of aphakic CLs. CL use requires specialist optometrist support, parental insertion and removal, and access to clean water. Pseudophakia is thus particularly attractive in lower income settings, where childhood cataract carries significant burden. However, the higher rate of re-operation suggests that IoL implantation is not a ‘one-stop’ solution for children in these countries. In addition, pseudophakic children will still require refractive correction for near focus, or if there has been a ‘refractive surprise’.

Advocates for paediatric IoLs also posit a positive impact on family well-being thanks to the avoidance of CL use. However, the IATS found no evidence of this, and instead found a temporary increase in parental stress with infant pseudophakia during the first year after surgery, which may have been related to the higher incidence of re-operation versus the aphakic group [46].

It is important to note that due to the rarity of CC and the resulting inability to subgroup patients into smaller, more homogenous groups, all studies are limited to some degree by having to cluster results and outcomes from infants with various types of cataract, associated ocular anomalies and underlying cause. For this reason, variation in outcome is commonly seen and reported.

CLINICAL TIP: A combination of high re-operation rates and sub-optimal refractive outcomes have curtailed IOL implantation in under 2-year olds in the UK. The authors usually plan for IOL implantation in children over the age of 2 years.

#### When to operate?

Managing dense infantile CC involves balancing the risks of general anaesthesia and secondary glaucoma (caused by early surgery) with irreversible deprivational amblyopia (caused by delayed surgery). The start-point and duration of the critical window within the sensitive period of early childhood visual development remains unclear. Age at surgery is the most important determinant of visual outcome: the later the surgical intervention, the worse the visual outcome [5, 47, 48]. There is conflicting evidence on the timing of a ‘cut off’ age at surgery at which outcomes suddenly worsen: it may be at 3–4 weeks of age [49, 50], 6 weeks [48], nearer 7–8 weeks of age [51, 52] or a ‘cut off age’ may not exist [5, 47]. The highest-level evidence we have suggests that for CC every additional week of age slightly reduces glaucoma risk but slightly increases the risk of deprivational amblyopia [5, 47]. This highlights the importance of identifying affected babies as early as possible through, for example, neonatal screening programmes, giving families and clinicians adequate time to reach clinical decisions.

CLINICAL TIP: Currently, in the UK, when operating on dense unilateral cataract in infancy, most surgeons would plan to perform surgery between 6 and 8 weeks of age and for bilateral cataract between 6 and 10 weeks of age.

## During surgery

### Basic surgical techniques

Cataract surgery in children requires general anaesthesia. It should be preceded by examination of both eyes under the same anaesthesia (EUA). Biometry (keratometry and contact or immersion A-scan ultrasonography) is performed intra-operatively in younger children, and pre-operatively using non-contact biometry (e.g. IOL Master) in older children to determine the IOL or CL power [53, 54]. Surgical technique is dependent on the age of the child, and whether IOL implantation is planned. The lens can almost always be aspirated or removed with a vitrectomy cutter. A phaco hand piece is not needed. Two small corneal/limbal incisions (20 or 23 gauge) are made. Trypan blue is usually used to stain the anterior capsule with an additional benefit of stiffening the capsule, aiding capsulorrhexis. Due to low scleral rigidity in children, there is a tendency for the AC to collapse. Incisions are thus not extended unless necessary.

Viscoelastic agents (typically Sodium Hyaluronate or Chondroitin Sulphate or a combination) are used to maintain the AC during capsulorrhexis and IOL insertion. Higher viscosity (cohesive) agents are often preferred to visco-dispersive agents.

AC depth is maintained with a balanced salt solution infusion (to which adrenaline or heparin may be added) via either a self-retaining AC maintainer cannula, or a handheld bimanual irrigation/aspiration cannula.

When IOL implantation is not planned, a typical approach is anterior capsulotomy via a manual capsulorrhexis technique or with a vitrectomy cutter. This is followed by aspiration of the lens matter, posterior capsulotomy and anterior vitrectomy. If IOL implantation is planned, manual anterior capsulorrhexis is usual, although automated vitrectorrhexis is preferred by some. In a survey conducted in 2003 by AAPOS, more than 50% of respondents worldwide preferred this combination: a vitrector for very young patients and manual anterior capsulotomy for older children [55, 56]. Some surgeons “polish” the lens capsule in an attempt to minimise lens epithelial cell re-proliferation [57, 58]. 23g vitrectomy is now most commonly used, enabling aspiration of adherent lens matter through smaller incisions [59]. However, 20g vitrectomy cutters may still have a role for cases with very dense cataracts or capsular plaques. The central posterior capsule (PC) must be removed in young children (up to 5–7 years) due to the significant risk of re-opacification. This may also be considered in older children with developmental delay where out-patient YAG capsulotomy may not be feasible [60–62]. If an IOL is to be implanted, the corneal wound is enlarged to 3.5 mm prior to insertion of the lens using either a folding or injecting technique. A commonly used approach is manual posterior capsulorrhexis, followed by anterior vitrectomy and insertion of IOL into the capsular bag. Alternatively, an IOL can be implanted into an intact bag, corneal wounds closed, and then pars plana posterior capsulotomy and anterior vitrectomy performed (as in the IATS study) [34]. Other techniques include intracapsular IOL insertion, followed by tilting up of the IOL and automated posterior capsulotomy and anterior vitrectomy. Optic capture, a deliberate positioning of the IOL optic through the posterior capsulotomy, provides great stability, and reduces PC opacification. If integrity of the anterior and/or posterior capsulorrhexis/capsulotomy is compromised, it may still be possible to place a three-piece IOL in the ciliary sulcus. Optic capture is advisable in such cases [63, 64] (discussed in more detail below). In a worldwide survey of 329 paediatric cataract surgeons conducted in 2007, the AcrySof hydrophobic acrylic IOL (Alcon Laboratories, Inc.) was the preferred implant (1-piece AcrySof IOL for in-the-bag implantation, and 3-piece for sulcus fixation. Multifocal IOLs are rarely used in children because they require precise refractive outcomes for optimal results. The refractive shift that occurs in growing eyes makes this impossible to predict. However, this may change as technology evolves [65, 66]. Intracameral triamcinolone [67–69] can be used to visualise vitreous strands, but when used, it is important to remove it, to minimise post-operative pressure spikes.

Before closure many surgeons perform a surgical iridotomy (with the vitrector) in eyes left aphakic after instillation of miochol (pilocarpine) to miose the pupil. In every case all wounds are sutured, typically with 10/0 polyglactin (Vicryl) [70]. This is followed by an intracameral injection of cefuroxime [71] and subconjunctival or intracameral steroid. Some surgeons leave a CL in place to correct the aphakic refractive error at the end of surgery. Most fit CLs 1–2 weeks post-operatively [53, 72, 73].

CLINICAL TIP: A consistently reliable bimanual technique is recommended for most CC. However, affected eyes can vary significantly and thus surgeons should be comfortable with a variety of techniques in order to achieve optimal outcomes.

### Managing CC associated with persistent foetal vasculature (PFV)

PFV, or persistent hyperplastic primary vitreous, is an important cause of unilateral and, occasionally, bilateral CC. The foetal hyaloid artery enters the developing optic cup through the optic fissure, travelling through primary vitreous to envelop the developing lens. Regression is normally completed by term. Failure of regression is incompletely understood and various genetic and teratogenic factors have been implicated [74].

**Fig. 3:**
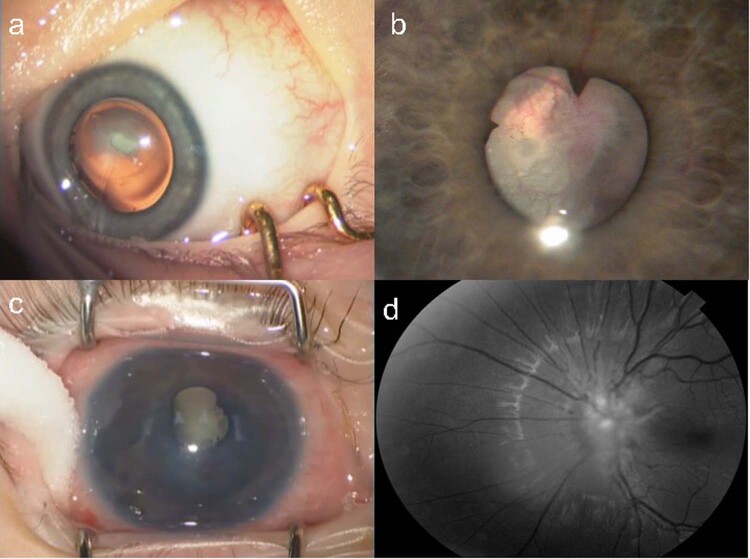
Persistent Foetal Vasculature (PFV). **a** Elongated ciliary processes can be seen inserting directly into the peripheral lens capsule. **b** Abnormal iris vasculature with intra-lenticular vasculature and irido-hyaloid remnants adhering to anterior lens capsule. **c** Secondary angle closure and buphthalmos in severe PFV. **d** Posterior PFV with mild tractional changes visible on retinal imaging.

PFV is classified as anterior, posterior or mixed. It is associated with cataract, microphthalmia, microcornea, ciliary processes elongation (Fig. 3a), aberrant iris vasculature (Fig. 3b), shallow AC (Fig. 3c), intra-lenticular or capsular blood vessels and corneal opacity. Posterior hyaloid remnants can consist of a thin avascular remnant or thick fibrovascular stalk with patent blood vessels. Posterior PFV may feature retinal traction (Fig. 3d) with accompanying partial or total retinal detachment.

Most unilateral cases arise sporadically in otherwise well infants and are thus not investigated. However, bilateral cases (and rarely unilateral cases) may be associated with systemic, genetic and neurological disorders and warrants exploration of an underlying cause.

B-scan ultrasound reveals the extent of posterior segment involvement while Doppler imaging can demonstrate blood flow within hyaloid remnants [75, 76]. Infants with suspected PFV should thus undergo B-Scan ultrasonography before surgery to exclude other vitreous or retinal pathology.

Lensectomy is carried out for visual rehabilitation or to prevent or treat secondary angle closure glaucoma (SACG). Small, non-axial opacities or those with severe posterior segment involvement may not benefit from surgery. Severe traction with central dragging of ciliary processes and shallow AC may be at risk of SACG requiring urgent lensectomy.

Limbal or pars plana surgical approaches both have their advocates in PFV. A comparison of the two techniques found no difference in outcomes or complications [77]. However, a pars plana approach may increase the risk of inducing retinal detachment [78]. The authors thus operate via a limbal approach for the majority of PFV-related cataracts.

**Fig. 4:**
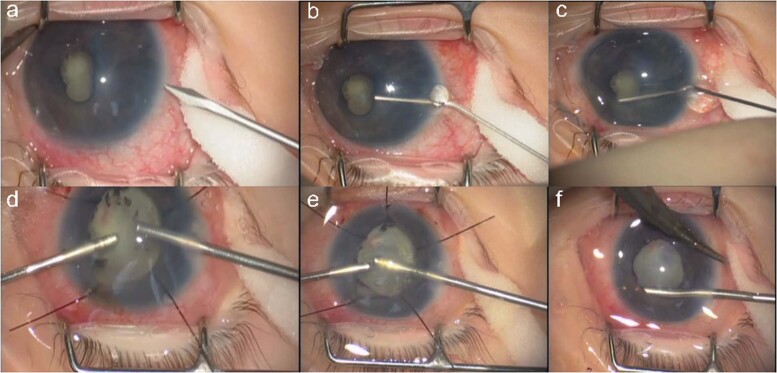
Surgical techniques for PFV related cataracts in children. **a** Limbal approach to surgery for SACG in PFV. **b** Use of high viscosity viscoelastic to reform AC. **c** The breakdown of pupillary adhesions is facilitated by viscoelastic. **d** Vitrectorrhexis undertaken with vitrector cutter. **e** Lensectomy performed bimanually. **f** Surgical iridectomy performed with low cut rate and 23g vitrector.

Surgery for mild PFV is similar to standard lensectomy. AC reformation may be challenging due to a tight fibrovascular-posterior capsular (FV-PC) complex pushing the lens forward. High viscosity, cohesive viscoelastics may assist and provide a degree of endothelial protection (Fig. 4a, b). Irido-hyaloid remnants or persistent membranes adhering to the anterior lens capsule require visco-dissection (Fig. 4c). Mechanical capsule vitrectorrhexis followed by lens aspiration may be preferred (Fig. 4d, e). Capsule staining with trypan blue can be helpful but in cases with long-standing kerato-lenticular adhesion, corneal endothelial staining can worsen the surgeon’s view [79]. A peripheral iridotomy can prevent pupil block and iris bombe from secondary pupillary membrane formation (Fig. 4f).

**Fig. 5:**
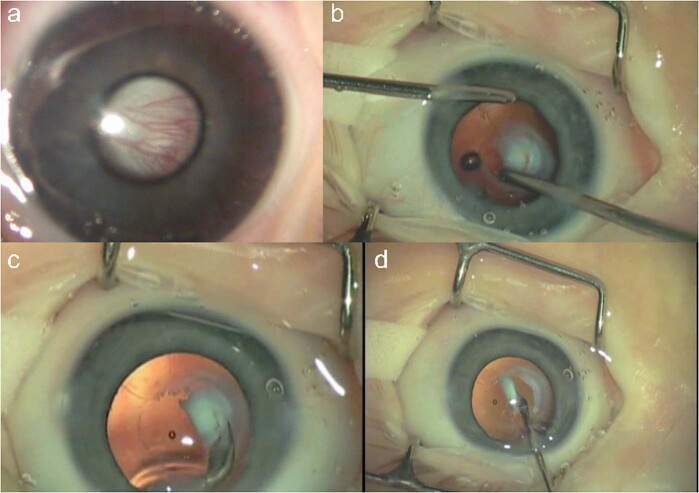
Lensectomy in PFV associated cataracts. **a** Vascularised posterior fibrovascular-posterior capsule complex. **b** Thickened capsule resistant to cutting by vitrector cutter. **c** Intraocular scissors used to open the capsule. **d** Intraocular diathermy used to cauterise the hyaloid stalk prior to truncation and anterior vitrectomy.

Patent blood vessels running within the FV-PC complex may require intraocular diathermy before FV-PC opening (Fig. 5a). The FV-PC complex can be thickened and may require a combination of narrow gauge microvitrectomy blade, intraocular scissors and vitrector (Fig. 5b, c), to open, avoiding excessive traction on the FV-PC complex and reducing the risk of an intraoperative retinal detachment. Where there is severe elongation of ciliary processes, intraocular scissors can be used to detach them from the FV-PC complex relieving traction. Radial incisions into peripheral capsule can remove circumferential traction but care must be taken to avoid cutting ciliary processes. The hyaloid stalk may need cauterising during the removal of the PC and anterior vitrectomy (Fig. 5d). Use of the Fugo plasma blade has been described to assist with this [80].

Whilst IOL implantation may be possible in mild PFV, re-operation rates are higher than with lensectomy alone [5, 34]. Early complications include hyphaema, vitreous haemorrhage, pupil block, iris bombe, SACG, corneal decompensation, peri-operative retinal detachment and post-operative hypotony. Later complications also include glaucoma, visual axis opacification, chronic hypotony and phthisis.

Anterior PFV has a more favourable visual prognosis than posterior or mixed PFV. As with other forms of CC, age at surgery, compliance with refractive correction and occlusion therapy, and development of glaucoma influences final visual outcomes (see Table 1).

**Table 1:** Visual outcomes following persistent foetal vasculature (PFV) associated cataract surgery.

Author	Year	No. of eyes	Mean age at surgery (Mo. ± SD)	Anterior PFV only (%)	Posterior PFV only (%)	Mixed Pfv (%)	Follow-up (Mo. ± SD)	+IOL (%)	% eyes non-quantifiable or NPL	% F + F/quantifiable VA	RD (%)	Vitreous haemorrhage (%)	VAO
Bawa Dass and Trese [81]	1999	27	16.4 ± 42.2	2 (7.4)	10 (37.0)	15 (55.6)	35 ± 37.1	0	70.4	29.6	16	12	0
Alexandrakis et al. [82]	2000	30	16	2 (6.7)	2 (6.7)	26 (86.7)	32	2	26.7	73.3	10	0	0
Anteby et al. [83]	2002	89	14.4 ± 22.8	29 (32.5)	28 (31.4)	30 (33.7)	75.6 ± 68.4	49.2	26.7	73.3	14.8	0	27.9
Sisk et al. [77]	2010	70	3.83	26 (37.1)	7 (10)	37 (52.9)	47	8.6	30	70	21.4	0	12.9
Morrison et al. [84] (IATS)	2011	18	2		0		12	38.9	0	94.5	5.6	11.1	22.2
Vasavada et al. [85]	2012	33	6.30 ± 5.16	33 (100)	0	0	36 ± 2	48.5	45.5	54.5	3	9.1	18.1
Kuhli-Hattenbach et al. [86]	2016	19	5.47 ± 3.47	16 (84.2)	0	3 (15.8)	53.21 ± 49		36.8	63.2	15.8	52.6	Not reported
Solebo et al. [87] unilateral (IOLu2)	2016	46	2.25	22 (47.8)	0	23 (50)	12	35	10	90	0	0	Not reported
Solebo et al. [87] bilateral (IOLu2)	2016	12	4.75	6 (50)	0	6 (50)	12	33	0	100	0	0	Not reported
Karacorlu et al. [88]	2018	44	3	5 (11)	5 (11)	34 (78)	37.2 ± 38.1	2	48	32	14	0	9

CLINICAL TIP: PFV-related cataracts often occur in association with additional ocular anomalies. Overall, they have a poorer visual prognosis. They can also have extra-ocular associations (particularly bilateral cases). Identification of PFV in children with CC guides pre-operative investigations and surgical management.

## After surgery

### Post-operative eyedrop regimes in paediatric cataract surgery

**Fig. 6:**
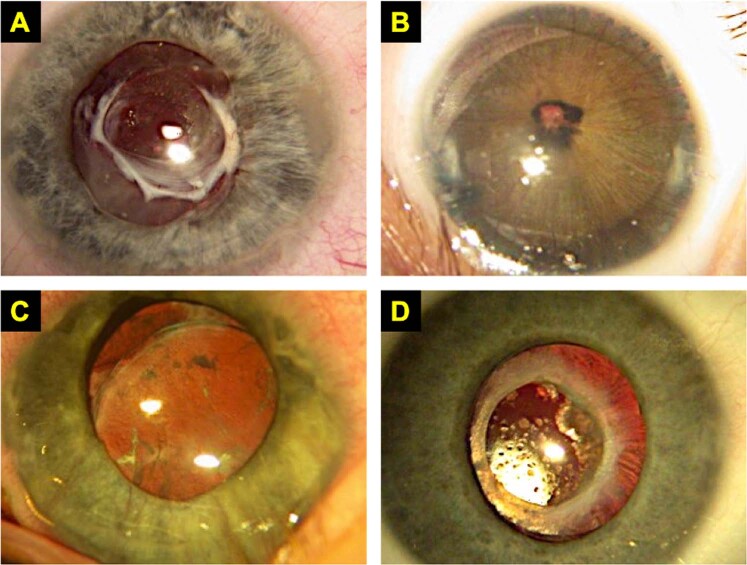
Sequelae of uncontrolled post-operative inflammation in paediatric cataract surgery. **a** Fibrinous membrane in juvenile idiopathic arthritis-related paediatric cataract. **b** Pupil block in a microphthalmic eye post lensectomy. **c** Tilted and subluxated intraocular lens implant and **d** deposits on intraocular lens implant with proliferation of lens epithelial cells.

Post-operative inflammatory responses in young children are more vigorous than in older children and adults; they are particularly strong in infants and in those with uveitic cataracts. This can lead to pain, pupillary membrane and posterior synechiae formation, pupil-block glaucoma, and IOL deposits and decentration (Fig. 6) [89]. Post-operative endophthalmitis in children has a poor outcome. The aim of post-operative drop use following cataract surgery in children is therefore to minimise inflammation and, in conjunction with intraoperative antibiotics, to reduce the risk of infection.

Eyedrop use in children presents specific challenges. Regimes must be effective and practical to maximise compliance in reluctant children, and minimise the risk of local and systemic side effects of topical corticosteroid therapy [90]. Any young child on long-term topical corticosteroids should be referred to paediatric endocrinology for assessment of adrenal suppression.

#### Corticosteroids

Topical corticosteroids are routinely used in a tapering dose regimen, usually over 4–6 weeks. Dexamethasone is most commonly used; stronger preparations, such as prednisolone acetate, are typically reserved for children at risk of more severe inflammatory responses. Oral steroids are usually unnecessary if a careful surgical technique and per-operative steroids have minimised any inflammatory response.

#### Side effects of corticosteroids in children

Up to one fifth of children develop raised intraocular pressure, with onset at weeks or months after starting topical steroids [91, 92]. Frequent topical steroids can also lead to growth suppression [93], Cushing syndrome [94] and hypothalamic–pituitary–adrenal axis suppression [95], which in turn can cause adrenal insufficiency. The symptoms and signs of this include: failure to thrive, weakness, hypotension, hypoglycaemia, nausea, vomiting and adrenal crises. A recent study of 26 infants undergoing cataract surgery under the age of 2 years identified adrenal suppression in two-thirds of patients on topical glucocorticoids, and a significant association between the cumulative glucocorticoid dose and a pathological ACTH stimulation test response [96]. Two subjects developed Cushing syndrome and one had an adrenal crisis during general anaesthesia. Punctal occlusion following drop administration may reduce systemic absorption and is advised for infants prescribed topical steroids.

#### Topical antibiotics

The routine use of pre-incision topical povidone-iodine, in combination with intracameral antibiotics, is likely to have significantly reduced the risk of infective endophthalmitis in paediatric cataract surgery. Post-operative topical antibiotics are also routinely given, usually in combination with topical steroid.

Combined antibiotic/steroid drop combinations include dexamethasone 1 mg/ml, hypromellose 5 mg/ml, with neomycin (as neomycin sulfate) 3500 unit/ml, and polymyxin B sulfate 6000 unit/ml (Maxitrol), or betamethasone and neomycin 0.1% (betnesol-N, licensed for children over 2 years old). All contain benzalkonium chloride preservative.

Chloramphenicol, 0.5% or 1%, a broad-spectrum antibiotic, can be prescribed for patients requiring separate preservative-free antibiotic and steroid drops. Side effects of chloramphenicol, including bone marrow disorders, have been described but are extremely rare. It thus is commonly prescribed in the UK. Ofloxacin is licensed for children over the age of 1 year. Post-operative antibiotic drops are usually prescribed until corneal sutures are absorbed.

#### Cycloplegia

Cycloplegia following cataract surgery aims to avoid posterior synaechiae formation when inflammation is present, and to minimise discomfort. Cyclopentolate 0.5% (below 6–12 months) or 1%, twice or three times daily, for 1–2 weeks is usually prescribed. Atropine 1% once a day may be an alternative in older children. Phenylephrine 2.5% can be used with cyclopentolate if enhanced pupillary dilation is needed, for example following cataract surgery combined with pupilloplasty or anterior segment revision. Some young children demonstrate a hypersensitivity reaction following cyclopentolate use, with facial flushing, tachycardia and fever. If a patient has a history of cyclopentolate sensitivity, then tropicamide 0.5–1% may be used as an alternative.

#### Typical post-opoperative eyedrop regimes following cataract surgery in children

A combined antibiotic and steroid drop used initially 4–6 times a day, tapering over 4–6 weeks, is usually sufficient and acceptable to parents and children (Table 2).

**Table 2:** Suggested ‘standard’ drop regime following routine paediatric cataract surgery.

Medication	Frequency and duration
Topical steroid (e.g. dexamethasone 0.1%)	2 hourly—1 week 4 times/day—1–2 weeks Taper off over 4–6 weeks
Topical antibiotic (e.g. chloramphenicol 0.5%)	4 times/day—until corneal sutures dissolved (Not required if using a combination drop e.g. maxitrol i.e. in cases not receiving a contact lens)
Mydriatic (e.g. cyclopentolate 0.5 or 1%)	2–3 times/day—2 weeks
Combined steroid and antibiotic (e.g. maxitrol ointment)	At night—3 weeks

Separate antibiotic and steroid preservative-free drops can be used in children with preservative allergy, and those left aphakic using CLs after surgery. The standard drop regime may also need to be modified post-operatively in uveitic cataract cases, after iris hook use, following traumatic surgery or when there has been previous glaucoma surgery.

### Refractive correction after cataract surgery in children

The paediatric eye is left with significant hypermetropia following cataract surgery. This can be up to +30.00 DS in aphakic eyes but is less marked in pseudophakic eyes, although dependent upon the age of the child and the refraction aimed for.

**Fig. 7:**
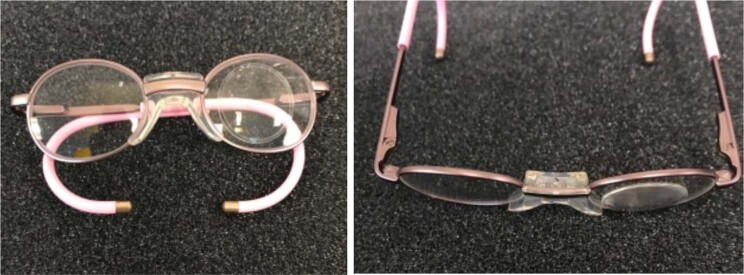
Spectacles fowllowing cataract surgery in children. Self-adhesive lenses can be applied to any spectacles to achieve high refractive correction.

It is important to correct any refractive error as soon as possible after surgery in order to provide a focussed retinal image and thus enhance visual development. High hypermetropic refractions mean that the required corrective spectacle lenses are very thick. Therefore, as small a frame size as possible should be used to reduce this. Many frame manufacturers now make small, stock frames for babies, that are sensibly priced and cosmetically appealing. The range in post-operative refractive errors, combined with variability in the sizes of frames required, precludes clinics from stocking an adequate selection of ready-made spectacles. Some clinicians use a system of stick-on, high-powered lenses (associated optical). The UniVision system is designed for the low vision market, but with their small diameter and high plus powers, these self-adhesive lenses can be stuck on to any frame to give an instant result, while the child’s refraction is stabilising, or while their permanent spectacles are being manufactured (Fig. 7).

**Fig. 8:**
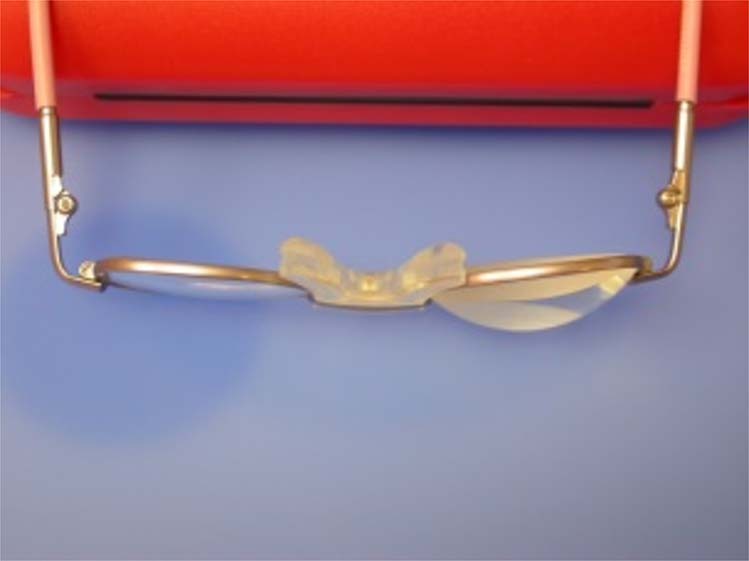
Unilateral aphakic correction in paediatric spectacles. Lenses tend to be heavy and can be poorly tolerated in children.

The high hypermetropia in paediatric aphakia is associated with poor optical properties in spectacles. The main refractive power is concentrated at the centre of the lens which leads to optical edge effects and a reduction in the peripheral field. The glasses tend to be heavy and are not well tolerated by some children (Fig. 8).

CLs offer a better solution in most cases, as the refractive power of the lens remains centred over the visual axis, no matter where the child is looking. In addition, in children who have had unilateral surgery, spectacles cause image size disparity (aniseikonia) between the phakic and aphakic (or even pseudophakic) eyes, which is minimised by CLs. This is an important factor in reducing the potential causes of amblyopia and encouraging binocularity development.

Soft, silicone hydrogel daily lens wear is usually the modality of choice. These lenses are stable in the eye, well tolerated and reasonably priced. The lenses are changed every 4–6 weeks, depending on how well they are looked after, and typically three or four pairs are provided at a time.

The first lens fitted depends upon the diameter and curvature of the cornea and the refraction of the eye. These parameters can be challenging to determine, but as a guide, the newborn to 6-month old cornea has an average radius of curvature (K) of about 7.10 mm or 47.59 D. The CL will have a high central thickness so a lens is fitted that is only slightly flatter in curvature than flattest K reading, as there will be minimal drape. A lens diameter about 1 mm wider than the corneal diameter, works well.

**Fig. 9:**
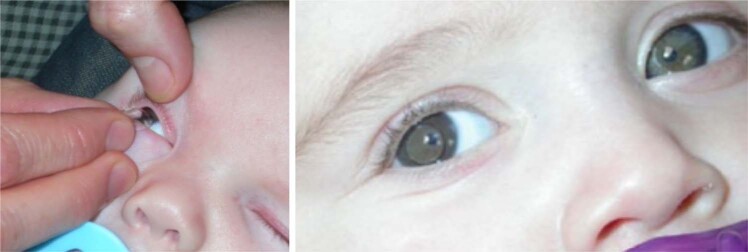
Contact lenses in young children. Technique for contact lens insertion.

To insert the lens, it is held between thumb and first finger to form a ‘petal’. It is then inserted by gently lifting the upper lid and ‘posted’ under the lid (Fig. 9). The lens should sit fairly centrally, so that the power ‘bump’ of the lens is over the pupil. The lens should move slightly with blinking or when pushed with the lids.

**Fig. 10:**
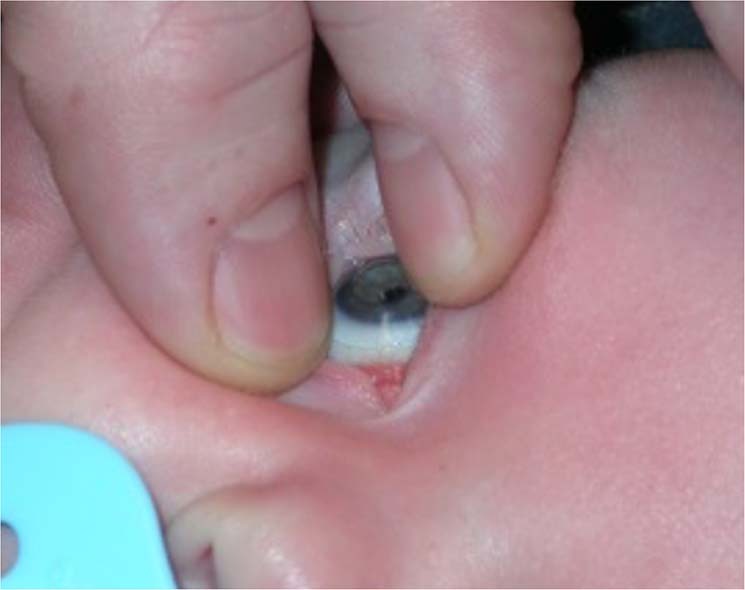
Contact lenses in uoung children. Technique for removal of paediatric contact lenses.

Children have smaller inter-palpebral apertures than adults, therefore the normal method of lens removal used in adults—by pinching the lens—is difficult to perform. Removal of the lens is more easily achieved by using the lids to squeeze the lens margins (Fig. 10).

In most cases, the first CL fitted is left in situ in for a week, before completing teaching the parents to insert, remove and care for the lens. Trying to get everything completed at the first visit can be stressful for both the parents and the child. At the first and all subsequent appointments, lens fit is checked and an over-refraction is carried out before the lens is removed. The eye is examined, a refraction carried out and IOPs measured. Parents are advised to remove the lenses daily and to replace them every 4–6 weeks. This routine is repeated every 3 months and lens specifications changed as necessary.

CLINICAL TIP: Refractive correction in aphakic and pseudophakic children can be as important as the surgery itself in achieving optimal visual outcomes. Services should ensure that they have access to specialised optometric teams experienced in the refractive correction in children following cataract surgery.

### Orthoptic management after paediatric cataract surgery

The Orthoptist will assess visual acuity, manage occlusion treatment, and help provide advice and support to families.

Measurement of uniocular, quantitative visual acuities should be achieved as early as possible. Standard forced choice preferential looking (FCPL) tests are used for pre-verbal infants, but even when FCPL results are encouraging, the long-term prognosis for visual outcome remains guarded in early childhood [97]. Crowded subjective optotype visual acuity tests are introduced as soon as the child is able to use them. The optical correction distance should be considered when selecting the testing distance. In addition to the measurement of visual acuity, observation of fixation and visual behaviour are important. The presence or absence of nystagmus should be documented at every visit.

Treatment of amblyopia in unilateral CC represents a significant challenge. The condition is highly amblyogenic, causing unilateral stimulus deprivation pre-operatively, and high anisometropia, complicated by absence of accommodation, post-operatively. In addition, strabismus will develop in most cases [98].

There is general agreement that occlusion therapy is vital to the visual outcome in unilateral CC, but the exact relationship between the amount of occlusion and the final visual acuity achieved remains uncertain. The IATS found that occlusion therapy of 4 h or more daily, over the first 4 years, led to better visual outcomes than occlusion of less than 2 h. However, there was significant inter-individual variability. The number of hours occlusion achieved in the first year, and in the next 3 years combined, each accounted for ~10% of variance in optotype acuity at age 4½ [99]. Occlusion is usually introduced in a graduated manner in the first 6 months because there is some evidence that prolonged occlusion in very early infancy could disrupt developing binocular function and thus reduce the potential for stereopsis [100]. Similarly there is some evidence that prolonged occlusion may affect the un-operated eye causing reduced amplitude pattern Visually Evoked Potentials and reduced contrast sensitivity function at high spatial frequencies [10, 101].

Compliance with occlusion may be challenging, Allen found that occlusion was abandoned before age four in 31% of patients [97]. Poor compliance with occlusion may relate to the presence of well-established low vision (and poor visual potential) as well as, of itself, being a cause of eventual poor acuity in the operated eye.

In patients with Fusion Maldevelopment Nystagmus Syndrome (FMNS), the nystagmus initially worsens with occlusion, but there is evidence that it can stabilise after a period of adaptation to the patch [102]. The aim should thus be for patients with FMNS to occlude for longer each day, but for fewer days per week to maximise this stabilisation effect.

Current practice for unilateral CC is to start occlusion at 1 week post-operatively, provided there is a clear visual axis, and adequate optical correction is in place—usually initially with glasses until the eye has healed sufficiently for a CL to be used. Occlusion starts at 1 h per day for each month of life, until 6 months old. After this, 6 h per day is recommended and continued until at least 5 years of age. The amount may be modified, depending on the visual acuity results achieved. Some occlusion is usually continued until age seven but the amount may be reduced once the child starts school, again depending on the level of acuity achieved [97].

In children with bilateral infantile cataract, occlusion should be started if an acuity difference (and/or fixation preference) is identified, visual axes are clear, and there is adequate optical correction. The occlusion times suggested will be individual to each patient based upon the visual acuity results. Not all bilateral patients will need occlusion.

In addition to the conventional orthoptic roles of assessing acuity, strabismus and nystagmus, orthoptists in many units also ensure that patients and their families are informed about and are able to access additional educational and visual impairment support required. In larger units this role is typically performed by an Eye clinic liaison officer. This input is particularly important in children who have had bilateral cataracts, but unilateral cases may also need support while occluding.

## Glaucoma following cataract surgery

### Incidence

Glaucoma following cataract surgery (GFCS) in children is noted whenever there is thorough follow-up and post-operative surveillance. Reported rates vary [103–110] from 2 to 58% (Table 3) with most reporting a 10–25% risk and a trend to higher rates with longer follow-up and younger age at surgery. These rates oblige surgeons to discuss glaucoma with families, including possible treatment pathways, before cataract surgery is undertaken. This may tip the balance against intervention in unilateral cases if significant predictors of adverse outcome are also present. Glaucoma can occur decades after childhood cataract surgery and surveillance should thus continue for life [107].

**Table 3:** Glaucoma rates following paediatric cataract surgergy. Published series of GCFC (glaucoma following cataract surgery).

	Rate (by eye not patient)	*n* (eyes)	Follow-up duration	Date	Locality
Chen et al. [103]	58%	368	10.3 years mean	1970–2003	Massachusetts, USA
Lundvall and Kugelberg [105]	12%	137	9.6 years mean	1980–1997	West Sweden
Tatham et al. [106]	2%	104	6.4 years mean	1987–2009	Leicester, UK
Rabiah [104]	21%	570	9.0 years mean	1983–1996	Saudi Arabia
Vishwanath et al. [107]	16%	128	5 years end point	1994–1997	London, UK
Michaelides et al. [109]	21%	71	5 years end point	1994–2000	London, UK
Chak et al. [110]	10%	275	6 years minimum	2002–2003	UK
Solebo et al. [108] IOLu2	10%	221	12 months end point	2009–2010	UK and Ireland

### Definition and case identification

Glaucoma in childhood is defined as two or more of the following: raised intraocular pressure, optic disc cupping, visual field defect, corneal changes (Haab striae or enlarged diameter) or globe enlargement [111].

GFCS can be subcategorised according to whether the angle is open or closed [111]. Following surgery with modern instruments most presentations of GFCS are in the context of a deep AC and, not uncommonly, with corneal oedema. Rebound tonometry under-estimates IOP in these circumstances and a normal apparent IOP value in this context does not exclude glaucoma.

### Risk factors

Clinical series [104, 106–109] have shown that younger age at surgery confers higher glaucoma risk. Surgery before the age of 4 weeks increases the risk of glaucoma fourfold [107] with some reports finding that glaucoma only occurred after surgery below a threshold age; between 6 [108] and 9 [104] months with an ~2% reduction in risk for each extra week of age at surgery [108].

The pathophysiology of GFCS remains incompletely understood. Hypotheses largely explore potential causes of impaired aqueous drainage and include insult to or altered function of the trabecular meshwork. Liberated lens epithelial cells [112, 113], vitreous factors [109], steroid exposure, inflammation, mechanical trauma or altered zonular forces have all been implicated.

Many ocular pathologies and surgical procedures co-occur with chronic GFCS and hence have been reported as associations; examples include primary posterior capsulotomy [109] (hazard ratio, HR = 10.7) [104], secondary membrane surgery (HR = 2.6) [104], microcornea [103] (HR = 1.9) [104], pupil block [105], residual lens material [114], post-operative pharmacological dilation [103], post-operative complications [103, 110] and micro-ophthalmia [110]. However, co-occurrence does not necessarily confer aetiology and it has been difficult to remove cofounding variables from analysis to better understand causality, in particular the influence of a child’s age at the time of surgery.

For example, implantation of IoL is technically easier in older infants with larger eyes, and so is performed more often in this group who are also less likely to develop glaucoma. IOLs were speculated to be protective against glaucoma, but this has been shown not to be the case after statistically controlling for age [5, 39].

### Timing of surgery

Surgery for dense cataracts has to be performed before the ‘critical period’ for vision has concluded, to avoid intractable amblyopia. This critical period has been determined to be as short as 6 [115] to 7 [116] weeks. Birch et al. suggested a bi-linear relationship in which sensitivity to visual deprivation continues to reduce until about 15 weeks of age [49]. Most authors agree that the optimum time for surgery for visually significant bilateral CC is at about 6–8 weeks corrected gestational age. Birch and Stager demonstrated a similar critical period for unilateral cases despite their higher amblyogenic nature [4].

### Management

Medical treatment can control GFCS for many years and is more likely to successfully delay surgery in those cases which are later-onset. A medical regime should be chosen which is safe, not too onerous, and cost-effective. Most glaucoma medications with the exception of Latanoprost are unlicensed for treating glaucoma in children. Before prescribing, this should be explained to parents and the rationale for the medication choice. See medicinesforchildren.org.uk for information for prescribers, patients and parents regarding the use of unlicensed treatments.

Robust evidence demonstrating the relative superiority of one pharmacological agent over another for GFCS is sparse in children (or adults). About half of children with GFCS respond to latanoprost. Side effects are infrequent and mild though it is prudent to discuss with parents a likelihood of iris darkening and lash lengthening with prolonged use; more of an issue in unilateral cases.

Topical beta blockers have similar average efficacy to latanoprost when used as monotherapy and again are well tolerated. Beta blockers are relatively contraindicated in children with asthma. In our experience the commonest scenario in which beta-blocker toxicity becomes apparent is when a child has a chronic cough which improves on cessation of the causative eye drop.

Topical carbonic anhydrase inhibitors are also well tolerated in children. Patients with a ‘sulpha’ intolerance (usually from an antibiotic) should be considered to have a potential cross-sensitivity to acetazolamide though even with this history a reaction is unlikely. Systemic acetazolamide is useful for short periods but associated anorexia or lethargy mean it is often not a poor long-term strategy. There are reported instances of acute renal failure from crystalline deposition [117] and acute anuria should prompt urgent cessation of treatment and a paediatric assessment.

Alpha-agonists; brimonidine and apraclonidine have a limited role. The risk of CNS suppression is higher in younger, lighter children, in whom the drug can cause unconsciousness and apnoea but drowsiness is a risk at any age and local irritation is a problem. Apraclonidine is generally safer than brimonidine with regard to the risk of CNS suppression and is used preferentially especially in younger children. However, for children under 24 months of age, apraclonidine use outside of an operating theatre is only with caution, and after documented and informed consent and after hospital-supervised first dosing.

There are many sensible topical treatment combinations. A reasonable topical treatment escalation, with progression to the next step in the context of inadequate pressures is given here:

(i) Latanoprost or timolol 0.25% monotherapy.
(ii) Combination dorzolamide/timolol preparation.
(iii) Dorzolamide/timolol combination plus latanoprost.

If this regime or similar is inadequate to control the pressure, further medication may temporise but cyclo-ablation or surgical intervention should be strongly considered. Half of GFCS cases require laser or surgical intervention [118]. Definitive treatment particularly in early onset glaucoma, often requires an aqueous shunt.

Angle surgery has a role in cases with open drainage angles. Efficacy of 360 degree ab-externo trabeculotomy was first demonstrated by using a suture placed in Schlemm’s canal before tightening to in-fracture the inner wall [119]. An illuminated-tip fibre-optic cannula allows the surgeon to know where the cannula is and avoid a sub-retinal passage. Full circumferential passage is not always possible and has been shown to be achieved less often in GCFCS than in most other forms of childhood glaucoma. Complete cannulation tends to lead to superior pressure control [120, 121], however, adequate pressure control can be achieved even with less than 360° passage [120]. Haemorrhage is a common occurrence, which often spontaneously resolves but in an infant’s aphakic eye, bleeding into the posterior segment can necessitate a core vitrectomy [122].

## Correction of paediatric aphakia using IOLs

There are a number of options available for secondary IOL implantation in children. Secondary implantation of a previously aphakic eye may become indicated due to CL intolerance, surface infections, impracticality of, or dissatisfaction with, CLs and/or aphakic glasses. The procedure requires careful planning.

Contraindications include:

1. Relative microphthalmia.
2. Active ocular disease (e.g. uveitis or glaucoma).
3. Poor visual prognosis (e.g. severe ocular malformation, long-standing retinal detachment).

### Pre-operative assessment

Pre-operative assessment should include detailed history and examination of the eye (before and after dilation) to assess the extent of capsular and iris support and any co-existing abnormalities. Corneal size and clarity, AC depth, maximal degree of pupillary dilation, posterior synechiae and Soemmering ring formation, uveitis (present or previous) and glaucoma should be noted. The biometry and operating notes of the initial operation should be examined (if available). Where there is doubt about feasibility of sulcus implantation, UBM should be considered.

### IOL power calculation

SRK II formula has been recommended [123] but SRK/T and Holladay II formulae showed least predictive error in a series of 117 eyes that included eyes <20 mm axial length [124]. Thirty-one children with secondary IOL under 3.6 years were found to have the least median absolute error with SRK II, SRK/T and Holladay I (1.23–1.30D) [125]. IOL master was deemed more accurate than contact biometry method under general anaesthetic (1.80 ± 1.40D vs. 2.43 ± 1.83D; *p* = 0.01) [123]. The authors usually use the SRK-T formula and a combination of SRK-T and Hoffer Q for short eyes.

### Treat pre-existing problems

Pre-existing glaucoma and uveitis should be well controlled before secondary IOL implantation is performed, although surgery in such cases should only be considered with caution.

### Surgical preparation

**Fig. 11:**
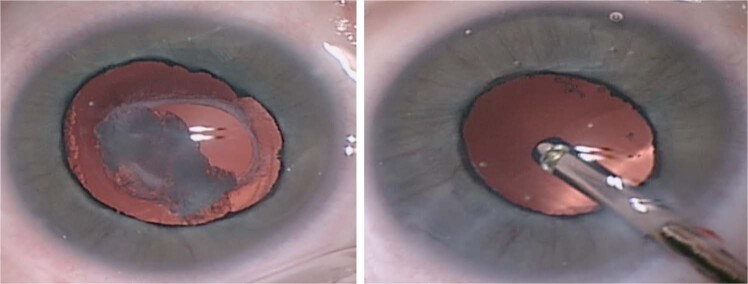
Surgical prepartion before secondary IOL insertion. An aphakic eye before and after removal of Elschnig pearls in the visual axis, moderate capsular phimosis, peripheral Soemmering and iridocapsular adhesions.

Miosis persisting despite prior mydriatics, can be managed via the use of viscoelastic, iris hooks or blunt viscosynechiolysis of any synechiae. Radial microsphincterotomies can also be useful. A Malyugin ring may be considered in older children but is not recommended in young children or small eyes. Pronounced miosis may require a surgical pupilloplasty with VR scissors or the vitrector. This will lead to permanent pupil dilation, useful for subsequent refraction and fundus examination (Fig. 11). Any pre-existing capsular phimosis, Soemmering ring formation or central vitreous membrane can be removed via a 20 G or 23 G vitrector in conjunction with anterior vitrectomy.

### Good capsular support

The presence of good capsular and zonular support enables a choice of techniques.

### Bag and sulcus fixation

Bag fixation requires opening of the fused leaves of the capsular bag with an MVR blade and removal of residual lens material (Nihalani and Vanderveen). However, if there is adequate sulcus space (demonstrated via EUA/UBM) ciliary sulcus fixation is a straightforward procedure. In sulcus implantation, it is essential to use either a foldable 3-piece or a PMMA IOL. A single piece AcrySof lens is not suitable for use in the ciliary sulcus. It will tend to decentrate and cause posterior iris chafing from its sharp edges. IOL power should also be reduced for sulcus fixation.

### Optic capture technique

**Fig. 12:**
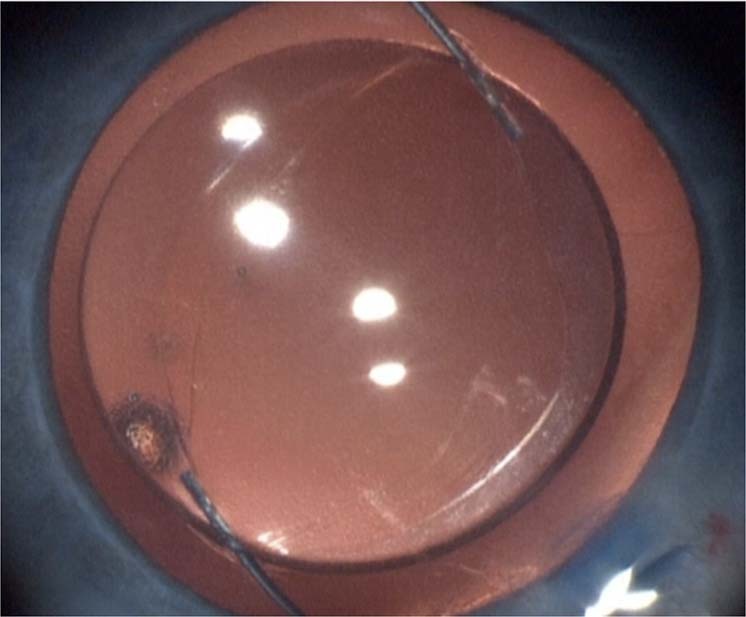
Optic capture. Optic capture advantages include centration and stabilisation of the IOL and counteraction of capsular phimosis and visual axis obscuration.

Optic capture can be considered when a three-piece IOL is implanted. This technique achieves long-term IOL centration and stability, prevents pupil capture, provides a vitreous barrier, inhibits capsular phimosis and visual axis obscuration by sequestrating the Soemmering ring peripherally (Fig. 12). Optic capture can be used in both bag and sulcus fixation with a choice of either capture of the optic behind the PC in bag fixation or behind the anterior and PC in sulcus fixation.

**Fig. 13:**
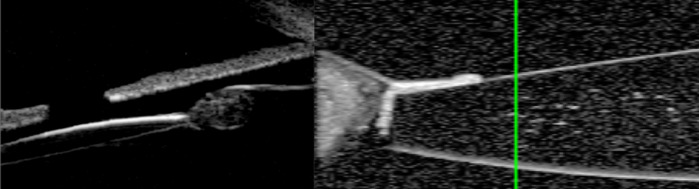
Optic capture. Optic capture sequesters lens cells and prevents visual axis opacity (VAO).

**Fig. 14:**
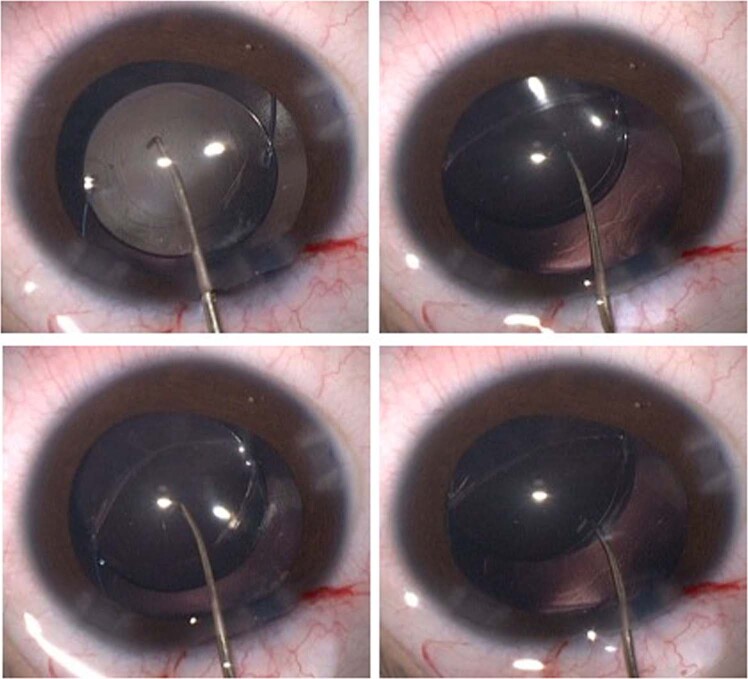
Optic capture of sulcus fixed IOL. Optic capture technique for IOL in the sulcus (a similar technique is employed for in the bag optic capture).

Optic capture sequesters proliferating peripheral lens epithelial cells via fusion of the anterior and PC, preventing the cells from migrating or proliferating into the visual zone (Figs. 13, 14).

### Bag-in-the-lens technique

**Fig. 15:**
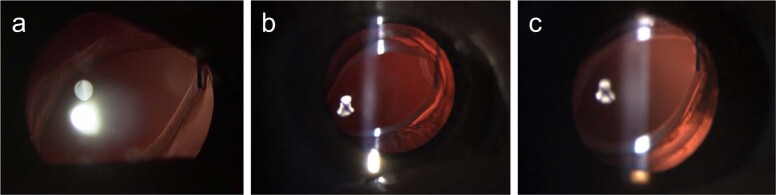
The visual axis is rarely obstructed after technically correct optic capture. **a** This child was implanted with combined anterior and posterior capture of the IOL optic and placement of the haptics in the sulcus. **a** 1 year post-surgery, **b** 2 years post-surgery, and **c** 7 years post-surgery.

The ‘bag-in-the-lens’ implantation technique developed by Tassignon can be used for both primary and secondary IOL implantation with excellent results reported [126, 127] (Fig. 15). The unique IOL design has an optic with a groove into which both the anterior and posterior capsulorhexis margins are placed. This technique has so far rarely been used in the UK.

### Poor capsular but good iris support

#### Anterior chamber IOL (AC IOL) technique

Morrison et al. implanted open loop AC IOLs in eight eyes of children (aged 5–17 years) with Marfan syndrome and found an average improvement of BCVA at ~1 year of 0.65–0.20 logMAR [128]. However, Epley et al. reported ten children with much longer follow-up (49.2 months). They described significant complications including pigmentary lens deposits, corectopia, haptic migration through the operative wound (requiring lens removal) and secondary glaucoma [129]. Long-term endothelial loss also remains a major concern and thus most of the authors do not use or recommend AC IOLs in children.

#### Pre- and retro-pupillary iris-claw IOL technique

In 1978, Jan Worst developed a PMMA iris-claw IOL. It is anteriorly vaulted to reduce risk of pupil block and pigment dispersion and designed to be enclavated into the relatively immobile midperiphery of the iris. Optic size is 5 mm with an overall diameter of 8.5 mm. It has no pointed haptics and a dioptric power range of +2 to 30 D. Over 300,000 such IOLs have since been implanted.

A review of 13 studies of 199 operated eyes with anterior iris-claw IOLs in 141 children reported few cases of pupillary block (eight cases), uveitis [7], retinal detachment [4], glaucoma [1] or endophthalmitis [1, 130]. However progressive endothelial cell loss following anterior fixation in children [131] has led to increasing use of a retro-pupillary fixation technique [132–135]. Concerns remain regarding long-term fixation stability and dislocation in paediatric eyes.

It is recommended that a peripheral iridotomy (with the vitrector) is performed when implanting an artisan IOL to prevent pupil block.

### Poor capsular support—with or without iris support

#### Transscleral sutured IOL

This has historically been a popular technique but high rates of IOL decentration due to suture degradation together with other vitreo-retinal complications, has led to it falling out of favour. The technique is now rarely performed in the UK in children.

#### Intrascleral sutureless IOL

Intrascleral haptic fixation is sutureless and therefore avoids many of the problems associated with sutured IOLs. It can be performed with [136] and without glue [137, 138]. Initial results appear reasonably promising:

Kumar et al. reported 41 eyes of 33 children aged 5–15 years who underwent glued intrascleral fixation via partial-thickness scleral flaps. BCVA > 20/60 was achieved in 46.3% of eyes. Complications included optic capture (2.4%), macular oedema (4.8%) and decentration (4.8%) with a mean endothelial loss of 4.13% at follow-up of 12–36 months.

Kannan et al. analysed 40 eyes of 25 patients (range 6–18 years) using a sutureless, flapless and glueless technique. A BCVA of ≥20/30 was reported in 85% of eyes. Early post-operative complications included hyphaema (10%), vitreous haemorrhage (2.5%) and ocular hypotony (2.5%). There were no apparent long-term complications. Follow-up ranged from 12 to 62 months.

Most recently a transconjunctival double needle technique has been described by Yamane et al. from Japan. This requires thin-walled 30 G needles (inner diameter 200 µm) to anchor flanged IOL haptics directly into the scleral wall without the need for scleral flaps, sutures or glue [134]. Adult results are very promising but as yet there are no published series reporting its use in children.

#### Post-operative care and complications specific to secondary IOL surgery

Immediate post-operative IOP spikes can be prevented by careful removal of the viscolelastic from the AC. Complex surgery and iris manipulation will typically result in more post-operative complications such as inflammation and ocular hypertension/glaucoma. The use of frequent topical steroid drops can minimise any uveitic response and topical anti-glaucoma medication can help with pressure spikes. Similarly, post-operative mydriatic drops are useful to prevent posterior synechiae formation. Frequent clinical reviews are advised to enable early detection of any complications.

CLINICAL TIP: Although technically challenging, there are now a range of surgical options available for secondary IOL implantation in children enabling successful long-term optical and visual rehabilitation. Consideration of IOL implantation in aphakic children, particularly those intolerant of CL wear or glasses, is an option at any age after early infancy.

## Summary

Paediatric cataracts are relatively rare, but a common and important cause of lifelong visual impairment. Visually significant CC require prompt assessment, diagnosis and surgical treatment in the first few weeks of life. Affected infants should be managed by specialised services with the expertise and infrastructure to achieve optimum outcomes. Significant advances in the field of genetics have dramatically changed the way children with CC are investigated, have led to a reduction in the number of investigations per patient, and increased the number of patients with a precise molecular diagnosis. These advances have also shown that cataract can be the presenting feature of a host of multisystem disorders in apparently well infants. Optimum outcomes for these children are often achieved only with early intervention. Improvements in surgical techniques and equipment have enabled visual outcomes from paediatric cataract surgery to be better than ever before. However, a good ophthalmic examination, and MDT work in combination with specialist optometric, orthoptic and other clinical colleagues remains crucial to achieving the best possible visual outcomes.
